# Interaction Between Arousals and Ventilation During Cheyne-Stokes Respiration in Heart Failure Patients: Insights From Breath-by-Breath Analysis

**DOI:** 10.3389/fmed.2021.742458

**Published:** 2021-12-16

**Authors:** Gian Domenico Pinna, Elena Robbi, Claudio Bruschi, Maria Teresa La Rovere, Roberto Maestri

**Affiliations:** ^1^Laboratory for the Study of Ventilatory Instability, Department of Biomedical Engineering, Montescano Institute – IRCCS, Istituti Clinici Scientifici Maugeri, Montescano, Italy; ^2^Sleep and Respiratory Function Unit, Montescano Institute – IRCCS, Istituti Clinici Scientifici Maugeri, Montescano, Italy; ^3^Laboratory for the Study of the Autonomic Nervous System, Department of Cardiology, Montescano Institute – IRCCS, Istituti Clinici Scientifici Maugeri, Montescano, Italy; ^4^Department of Pneumology, Montescano Institute – IRCCS, Istituti Clinici Scientifici Maugeri, Montescano, Italy

**Keywords:** hyperpnea, ventilatory overshoot, breathing instability, central apnea, apnea length

## Abstract

**Study Objectives:** Arousals from sleep during the hyperpneic phases of Cheyne-Stokes respiration with central sleep apnea (CSR-CSA) in patients with heart failure are thought to cause ventilatory overshoot and a consequent longer apnea, thereby sustaining and exacerbating ventilatory instability. However, data supporting this model are lacking. We investigated the relationship between arousals, hyperpnea and post-hyperpnea apnea length during CSR-CSA.

**Methods:** Breath-by-breath changes in ventilation associated with the occurrence of arousal were evaluated in 18 heart failure patients with CSR-CSA, apnea-hypopnea index ≥15/h and central apnea index ≥5/h. The change in apnea length associated with the presence of arousal during the previous hyperpnea was also evaluated. Potential confounding variables (chemical drive, sleep stage) were controlled for.

**Results:** Arousals were associated with a large increase in ventilation at the beginning of the hyperpnea (+76 ± 35%, *p* < 0.0001), that rapidly declined during its crescendo phase. Around peak hyperpnea, the change in ventilation was −8 ± 26% (*p* = 0.14). The presence of arousal during the hyperpnea was associated with a median increase in the length of the subsequent apnea of +4.6% (Q1, Q2: −0.7%, 20.5%; range: −8.5%, 36.2%) (*p* = 0.021). The incidence of arousals occurring at the beginning of hyperpnea and mean ventilation in the region around its peak were independent predictors of the change in apnea length (*p* = 0.004 and *p* = 0.015, respectively; R^2^ = 0.78).

**Conclusions:** Arousals from sleep during CSR-CSA in heart failure patients are associated with a rapidly decreasing ventilatory overshoot at the beginning of the hyperpnea, followed by a tendency toward a slight ventilatory undershoot around its peak. On average, arousals are also associated with a modest increase in post-hyperpnea apnea length; however, large increases in apnea length (>20%) occur in about a quarter of the patients.

## Introduction

Cheyne-Stokes respiration with central sleep apnea (CSR-CSA) is a common comorbidity and risk marker in patients with heart failure and reduced ejection fraction (1, 2). It predominantly occurs during stage 1 (N1) and 2 (N2) non-rapid eye movement (NREM) sleep (3) and is typically accompanied by a high number of arousals from sleep (3, 4). Despite the wealth of research conducted to elucidate its underlying mechanisms, some of them remain conjectural.

It is widely held that arousals from sleep occurring during the hyperpneic phase of CSR-CSA provide a transient extra-drive to the respiratory and upper airway muscles, thereby causing a transient ventilatory overshoot that adds to the ongoing, chemoreceptor-driven cyclic rise and fall in ventilation (5–9). This overshoot, in turn, would cause a greater hypocapnia, setting the stage for a new and longer central apnea. Thus, transient increases in ventilation induced by arousals would contribute to sustaining and exacerbating the ventilatory oscillation of CSR-CSA. The validity of this model, however, is uncertain, mainly because it is not possible to establish a clear causal link between arousal, ventilatory overshoot and increase in post-hyperpnea apnea length. Indeed, this link has been proposed based on results from observational studies (10, 11).

These studies, however, have some important limitations. First, they were performed in patients without heart failure. In the study by Xie et al. (10), for instance, patients had idiopathic CSA, a form of periodic breathing characterized by different underlying mechanisms, a much shorter hyperpnea length and arousal duration, and a different timing of arousal onset compared to CSR-CSA (4, 7, 12). Conversely, in the study by Khoo et al. (11), subjects were healthy volunteers with nocturnal periodic breathing induced by exposure to hypobaric hypoxia. Second, identification of arousal onset and offset, a crucial step in the assessment of the interaction between arousals and ventilation, was based on visual scoring of EEG activity (10, 11), which is often difficult and subjective. Third, the authors did not control for important confounding factors like chemical drive (10) and sleep stage (11).

Thus, the purpose of this study was to investigate the relationship between arousals and ventilation during hyperpneic phases of CSR-CSA in heart failure patients using (i) a computer-based approach for determining the time of occurrence and duration of arousals, and (ii) a case-control methodology to control for potential confounding variables. Moreover, we investigated the association between occurrence of arousals during hyperpneas and change in post-hyperpnea apnea length.

To address the first objective of the study, we followed a breath-by-breath approach. There are 2 reasons for this. First, during CSR-CSA arousals occur at the beginning of the hyperpnea or, more frequently, midway during its rising phase, and their duration commonly ranges from about 15 to 55% of hyperpnea length (4). Hence, arousals may affect ventilation during a limited portion of the hyperpnea that can be as short as the duration of 1 or 2 breaths. Second, since lung volume changes markedly during the crescendo and decrescendo phases of CSR-CSA, the ventilatory response to arousal might change as well on a breath-by-breath basis, owing to the concurrent change in the inhibitory feedback from lung stretch receptors (13, 14).

## Methods

### Subjects

We analyzed the polysomnographic recordings of 22 chronic heart failure patients who were part of an ongoing prospective observational study on the prevalence and clinical correlates of sleep apnea in heart failure. Inclusion criteria were: (i) moderate-to-severe heart failure (New York Heart Association class: II-III), (ii) reduced left ventricular ejection fraction (<45%), (iii) stable clinical condition and optimal medical treatment, (iv) dominant central sleep apnea with an apnea-hypopnea index (AHI) ≥15/h and a central apnea index ≥5/h. Exclusion criteria were: (i) myocardial infarction or cardiac surgery within the previous 3 months, (ii) chronic obstructive pulmonary disease, and (iii) use of sedatives (e.g. benzodiazepines, z-drugs and other GABAergic drugs, melatonin, trazodone and antidepressants with sedative properties) or therapy for CSA.

### Sleep Studies

Unattended standard polysomnography (15) was carried out between 9:30 p.m. and 7:00 a.m. in the patient's own bed using the portable polysomnograph Embla Titanium (Embla Systems, Thornton, USA). Sleep studies were visually scored according to the American Academy of Sleep Medicine recommendations (15, 16) using the Embla RemLogic software (Embla Systems, Kanata, Canada). Scoring information as well as raw signals were transferred to a dedicated workstation for subsequent analysis using dedicated software.

### Signal Analysis

Polysomnographic segments containing all episodes of CSR-CSA occurring during stages N1 and N2, were interactively selected. We used a previously validated methodology (4, 17, 18) to analyze the sleep structure and provide continuous information (resolution: 0.25 s) on the sleep-wake state of each subject within these segments. Arousals were automatically identified as any state change from NREM to wakefulness lasting ≥2 s, with the beginning and end of each change marking the arousal onset and offset, respectively (4).

Calibration of respiratory inductive plethysmography signals by the qualitative diagnostic calibration method (19) was performed by first selecting 3 5-min segments of regular breathing at the beginning, in the middle and at the end of the sleep study. The calibration factor was computed as mean value of the 3 calibration factors measured in the 3 segments, and was used to obtain a lung volume signal in relative units from rib cage and abdomen signals. Breath-by-breath tidal volume (*V*_*T*_), inspired minute ventilation (${\overset{∙}{V}}_{\text{I}}$) and inspiratory drive (*ID*) were computed as the difference between the end-inspiratory point and previous end-expiratory point, the ratio of *V*_*T*_ to breath duration, and the slope of the lung volume signal in the linear portion of each inspiration (20), respectively.

### Measurements

#### Breath-by-Breath Relationship Between Arousals and Ventilation

The relationship between arousals and ${\overset{∙}{V}}_{\text{I}}$, *V*_*T*_ and *ID* was investigated in each patient using a breath-by-breath, case-control, computer-based procedure. CSR-CSA cycles whose hyperpneic phase was preceded and/or followed by a hypopnea were excluded from analysis because the beginning and end of the hyperpnea may be ambiguous.

**Figure 1:**
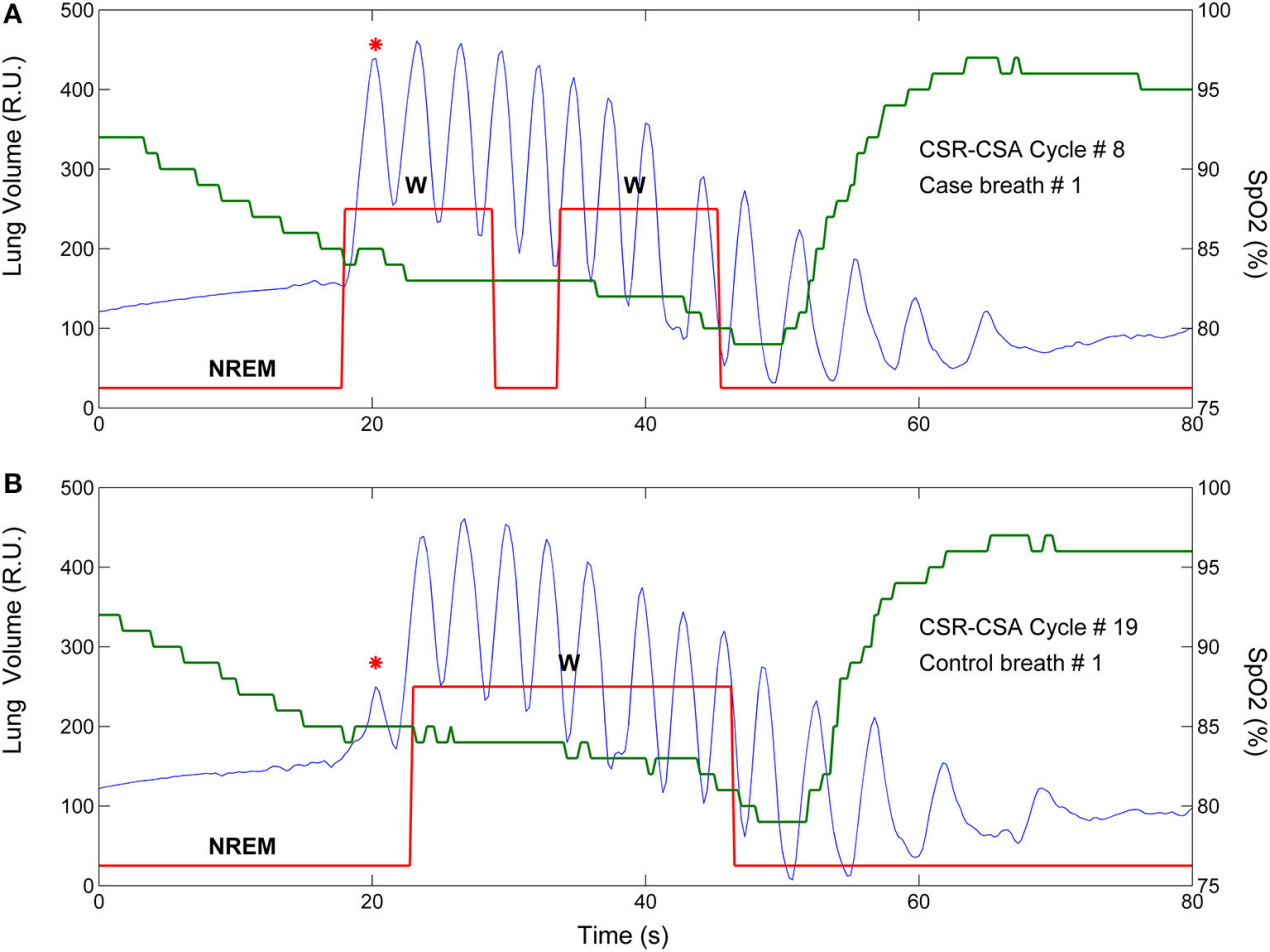
Example of breath-by breath case-control matching in the assessment of the relationship between arousals and ventilation. Blue solid line: lung volume; red solid line: state-transition diagram providing continuous information (resolution: 250 ms) on the sleep-wake state of the subject [stage 1, 2 non-rapid eye movement (NREM) sleep or wakefulness (W)] (4); green solid line: estimated oxygen saturation (SpO_2_) at carotid chemoreceptors. **(A)** An arousal occurred during the hyperpneic phase of the 8^th^ CSR-CSA cycle, potentially affecting several breaths (case breaths). A short (≈5 s) transition to NREM sleep during the arousal can also be noticed. **(B)** A matched control breath for the first case breath was found in the 19^th^ CSR-CSA cycle (closest cycle). This case-control pair is indicated by an asterisk. By definition, the two breaths had similar mean SpO_2_ (84.5 vs. 85.0%), similar duration of the hyperpneic phase (48 vs. 51 s) and same sleep stage (N2). RU, relative units.

A breath of the hyperpneic phase of a CSR-CSA cycle was classified as “case” if there was a temporal overlap between an arousal and the breath. For each case, a search was made for a matched “control” breath in the closest CSR-CSA cycle, according to the following criteria: (i) same sequential position (first, second, third…); (ii) no concurrent arousal and ≥10 s from the arousal offset of a previous arousal, if any; (iii) same estimated mean oxygen saturation at carotid chemoreceptors ±1.5% (see details below); (iv) same sleep stage (N1 or N2); (v) same hyperpnea length ±15%. If a control was found (Figure 1), the normalized arousal-associated change ($\hat{AAC}$) in ${\overset{∙}{V}}_{\text{I}}$, *V*_*T*_ and *ID* for *that case-control pair* was measured as the difference between the case breath and the control breath, normalized by, respectively, the average ${\overset{∙}{V}}_{\text{I}}$, *V*_*T*_ and *ID* in the absence of arousal during all hyperpneas, namely for ${\overset{∙}{V}}_{I}$:


(1) $$\begin{array}{l} \hat{AAC}\;{\left({\dot{V}}_{I}\right)}_{ijk}\\ =\frac{{\dot{V}}_{I}\;{\left(case\;breath\right)}_{i,j}-{\dot{V}}_{I}\;{\left(control\;breath\right)}_{i,k}}{Average\;{\dot{V}}_{I}\;in\;the\;absence\;of\;arousal\;during\;hyperpneas}\\ \times 100 \end{array}$$

where *i (i* = *1,2,3...N)* is the progressive number of the breath, *j* (*j* = 1, 2, …*M*) is the progressive number of the CSR-CSA cycle containing the case breath, and *k* (*k* = 1, 2, …*L*) is the progressive number of the cycle containing the control breath. Thus, for instance, ${\hat{AAC}\;\left({\overset{∙}{V}}_{I}\right)}_{3,20,22}\;$refers to the case-control pair including the third breath of the 20^th^ CSR-CSA cycle (case breath) and the third breath of the 22^nd^ cycle (control breath). Accordingly, if ${\hat{AAC}\;\left({\overset{∙}{V}}_{I}\right)}_{3,20,22}$ = 50%, the increase in ventilation in the case breath relative to the control breath is equal to half the average ventilation in the absence of arousals during hyperpneas. This computation was reiterated until all possible case-control pairs were identified in each patient across all CSR-CSA cycles. The median of $\hat{AAC}$ measurements obtained for each consecutive breath (first, second etc.) was then used as an estimate of the patient's arousal-associated change (AAC): ${\overset{∙}{V}}_{\text{I}}$ *V*_*T*_ and *ID* *for that breath*, provided that the number of case-control pairs was ≥5.

**Figure 2:**
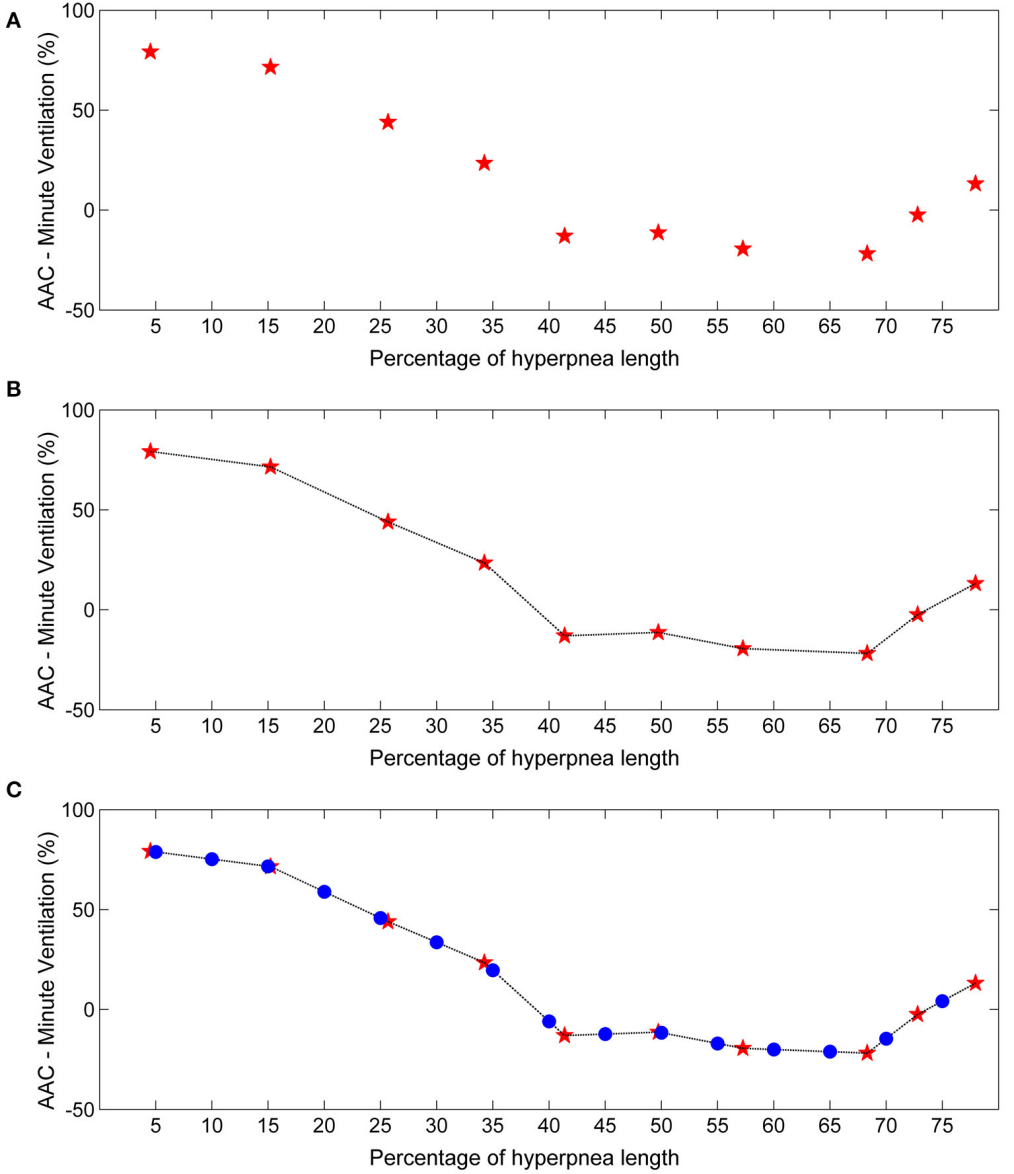
Example of estimation of the time course of the arousal-associated change (AAC) in minute ventilation. **(A)** Raw time course of the AAC. Red stars represent AAC estimates for each consecutive breath of the hyperpnea (first, second etc.) as a function of percentage of hyperpnea length. Percentages are reported up to 75% of hyperpnea length due to missing data in almost all patients (see details in the Results section). **(B)** Linear interpolation of AAC estimates. **(C)** The AAC was re-computed (blue dots) at predefined percentages of hyperpnea length (5, 10, 15%, ….) in order to compare the time course of the AAC among patients.

For each identified case-control pair, the time of end-inspiration of the case breath was measured and expressed as a percentage of hyperpnea length. The median of the measurements obtained for each consecutive breath (first, second etc.) was then used as an estimate of the time of occurrence of that breath. Using this information, the raw time course of the AAC in ${\overset{∙}{V}}_{\text{I}}$, *V*_*T*_ and *ID* was obtained. A representative example for ${\overset{∙}{V}}_{\text{I}}$ is given in Figure 2A.

In order to compare the time course of the AAC among patients, linear interpolation of individual AAC values was performed to obtain AAC estimates at predefined values of hyperpnea length (5, 10, 15%, ….) (Figure 2B). The result was a standardized plot showing the time course of the AAC during the hyperpnea (Figure 2C).

#### Estimation of the Time Course of Baseline Ventilatory Parameters

**Figure 3:**
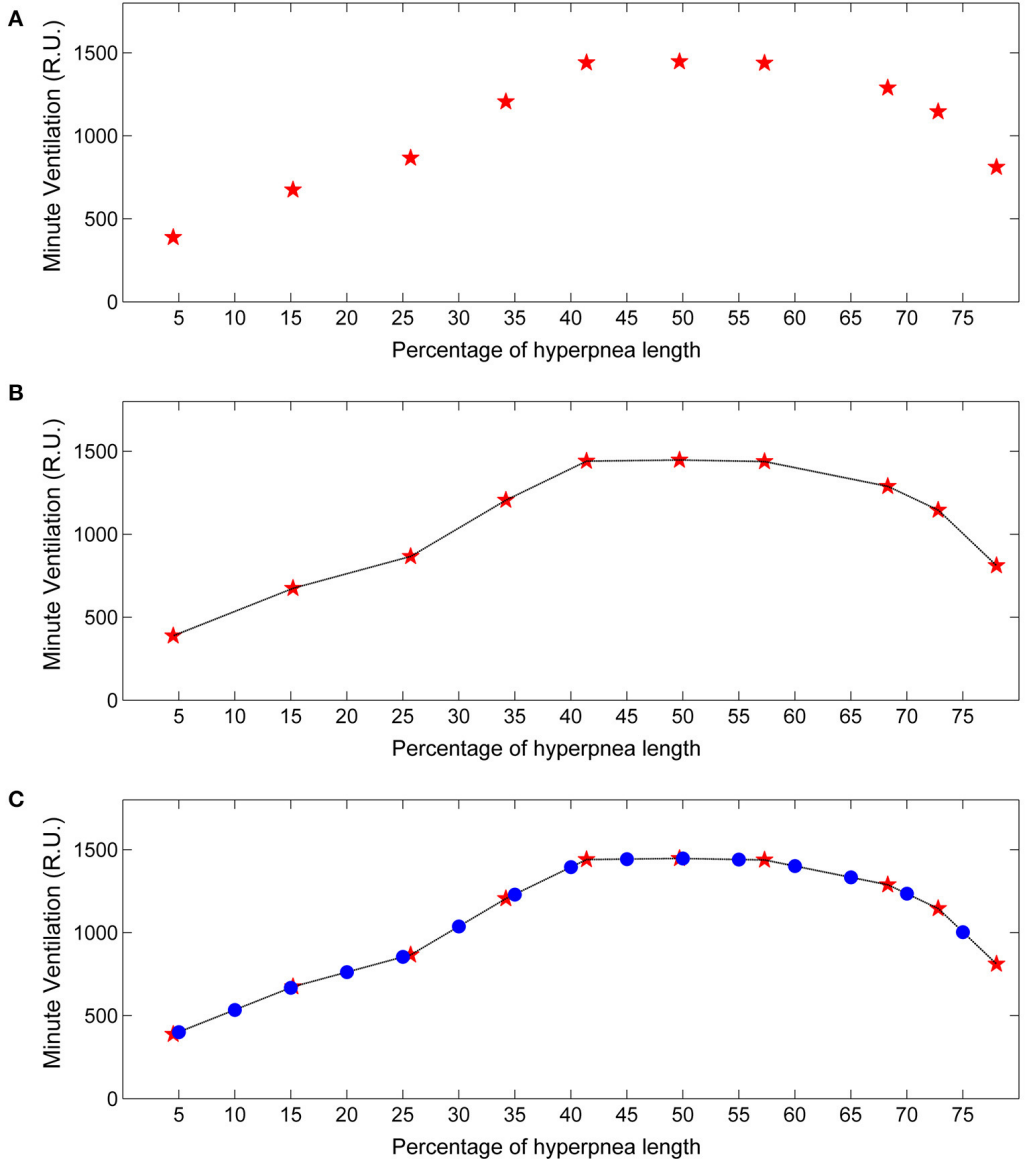
Example of estimation of the time course of baseline minute ventilation during hyperpneas. **(A)** Raw time course of minute ventilation in the absence of arousals (see text). Red stars represent minute ventilation estimates for each consecutive breath of the hyperpnea (first, second etc.) as a function of percentage of hyperpnea length. **(B)** Linear interpolation of the estimates. **(C)** Minute ventilation was re-computed (blue dots) at predefined percentages of hyperpnea length (5, 10, 15%, ….) in order to compare the time course among patients. RU, relative units.

For each identified case-control pair, the ${\overset{∙}{V}}_{\text{I}}$, *V*_*T*_ and *ID* of the control breath was also recorded. The median of the measurements obtained for each consecutive breath was then used as an estimate of the patient's baseline value of these ventilatory parameters. Using again linear interpolation and standardization of the time scale, the estimate of the time course of baseline ventilatory parameters was obtained (Figure 3). This curve can be interpreted as representing the *expected* ventilation in the case breaths had the arousal not occurred. The same procedure was followed for *V*_*T*_ and *ID*.

#### Estimation of Oxygen Saturation at Carotid Chemoreceptors

It is widely accepted that instability in the chemical feedback control system that regulates breathing is the primary underlying mechanism of CRS-CSA (5, 8, 9, 21, 22). Accordingly, cyclic hyperpneas occurring during CSR-CSA are caused by the concurrent cyclic stimulation of chemoreceptors by hypoxia and hypercapnia resulting from the delayed effect of previous apnea or hypopnea. Modeling studies and experimental observations indicate that, in the absence of arousals, carotid chemoreceptors exert a dominant influence on the development of periodic breathing (8, 23, 24). These receptors respond very quickly to changes in arterial blood gases (25). Accordingly, the rise and fall in ventilation during CSR-CSA would be almost synchronous with the rise and fall in arterial CO_2_ tension (PaCO_2_) at carotid chemoreceptors (8), and almost opposite in phase with rise and fall in arterial oxygen saturation (SaO_2_) at the same receptors (26).

Pulse oximetry (SpO_2_) is a commonly used technique for continuous monitoring of SaO_2_ in humans. In the context of sleep apnea, it is also often considered as a surrogate marker for the degree of cyclic asphyxia, on account of the occurrence of reciprocal changes in SaO_2_ and PaCO_2_ (27). The most appropriate site to monitor SpO_2_ during CSR-CSA is the ear, close to carotid bodies (26, 28). Indeed, the oscillation of SpO_2_ recorded with an ear probe is only slightly delayed relative to carotid bodies (29). However, monitoring of SpO_2_ during sleep studies is almost invariably performed using a sensor placed at the finger, as this site provides more stable recordings, is more comfortable for the patient, and allows an easier placement of the sensor. Because of the more distal position, changes in SpO_2_ at the finger are markedly delayed relative to carotid chemoreceptors (28).

**Figure 4:**
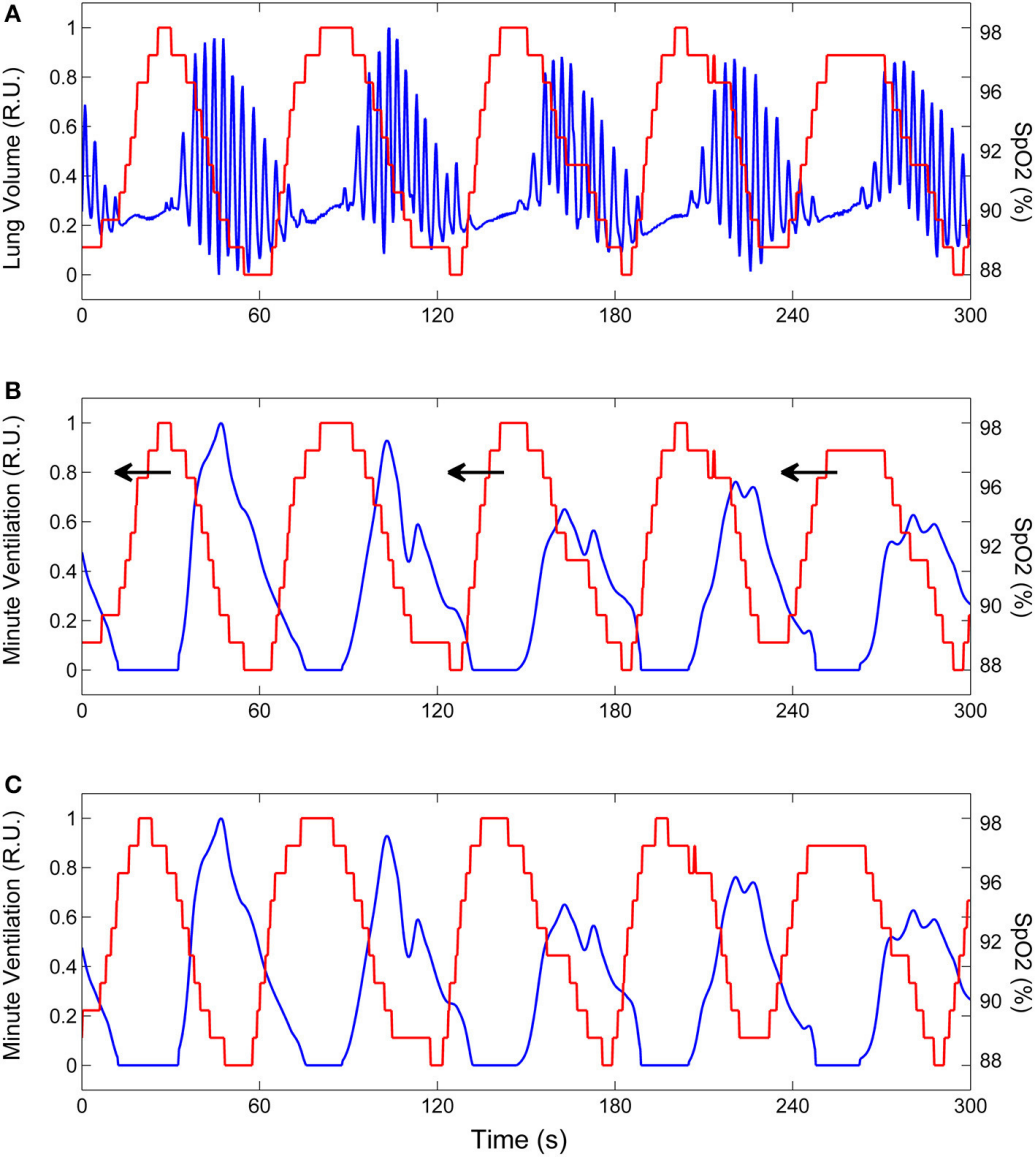
Example of the procedure to estimate the time course of oxygen saturation at carotid chemoreceptors. **(A)** Lung volume (blue line) and oxygen saturation at the finger (SpO_2_, red line) during CSR-CSA. **(B)** Same as **(A)** but the lung volume signal is replaced by the corresponding instantaneous minute ventilation signal. This signal was obtained using a previously developed method (29). To estimate the SpO_2_ signal at carotid chemoreceptors, the SpO_2_ signal at the finger was backward shifted in time as indicated by arrows. **(C)** Backward shifting was continued until the antiphase condition between the two signals was met (maximum value of the non-linear correlation coefficient between SpO_2_ and minute ventilation excluding apneas). The red line shows the estimated SpO_2_ signal at carotid chemoreceptors. RU, relative units.

Assuming, as outlined above, that during CSR-CSA ventilation is mainly driven by the cyclic activation of carotid chemoreceptors and that cyclic hyperpneas are almost in antiphase with cyclic desaturations, we estimated the time course of oxygen saturation at carotid chemoreceptors by shifting the SpO_2_ signal recorded at the finger backward in time, until the antiphase condition between hyperpneas and oxygen saturation was met. This condition was determined by finding the shift that maximized the value of the non-linear correlation coefficient between the SpO_2_ signal and the instantaneous minute ventilation signal during hyperpneas (28). The latter signal was obtained using a previously developed method (29). A representative example of this procedure is given in Figure 4.

#### Association Between Arousals and Post-hyperpnea Apnea Length

A case-control methodology was also used to assess the relationship between arousals and post-hyperpnea apnea length. An apnea was classified as “case” if it was preceded by a hyperpnea that contained one or more arousals. For each case, a search was made for a matched “control” apnea according to the following criteria: (i) absence of any arousal in the preceding hyperpnea, and (ii) same estimated mean SpO_2_ ±1.5% during the hyperpnea. This process led to the final identification of a set of independent case-control apnea pairs matched by similar mean SpO_2_ during the preceding hyperpnea. For each pair, the difference between the length of the case apnea and the length of the control apnea was computed. The association between arousals and apnea length was then estimated in each patient by taking the average of these differences, provided that the number of case-control pairs was ≥5.

### Statistical Methods

Depending on the distribution (normal or non-normal), we used the mean or median, respectively, as a measure of the central tendency of data across CSR-CSA cycles of each patient. These measures of central tendency were then used to compute group statistics. One-sample tests were performed using the *t*-test (normally distributed data) or Wilcoxon signed rank test (non-normally distributed data). The strength of the linear relationship between variables was assessed by Pearson's product moment correlation. We used multiple linear regression analysis to identify independent predictors of the change in post-hyperpnea apnea length. A *p* value < 0.05 was considered statistically significant and all tests were two-tailed.

## Results

The AAC could not be estimated in 1 patient because of the lack of breaths without concurrent arousal in some region of the hyperpnea, thus preventing the computation of the normalization factor in equation (1). In another 3 patients, the association could be estimated only in two-three breaths of the hyperpnea owing to an insufficient number (<5) of case-control pairs. These patients were excluded from further analysis. The mean age, New York Heart Association class and left ventricular ejection fraction of the remaining patients (*N* = 18) were 59.1 ± 9.8 years, 2.7 ± 0.5 and 28.4 ± 7.7 %, respectively. Sleep architecture and respiratory characteristics are reported in Table 1. Mean apnea, hyperpnea and cycle length of analyzed CSR-CSA cycles were 25.5 ± 6.3 s, 39.9 ± 7.9 s and 65.4 ± 11.7 s, respectively. The mean number of breaths per hyperpnea was 12.0 ± 2.2.

**Table 1:** Polysomnographic data of studied patients (*N* = 18).

**TST (min)**	**370 ± 52**
**N1 (% TST)**	**18.4 ± 6.0**
**N2 (% TST)**	**49.3 ± 9.3**
**N3 (% TST)**	**13.1 ± 8.8**
**R (% TST)**	**19.2 ± 4.4**
**Sleep efficiency (%)**	**79.8 ± 8.8**
**Arousal index (events/h)**	**24.8 ± 7.6**
**AHI (events/h)**	**40.4 ± 8.6**
**CAI (events/h)**	**21.5 ± 10.0**
**ODI (events/h)**	**45.0 ± 10.3**
**Minimum oxygen saturation (%)**	**83.1 ± 4.6**

*Continuous data are expressed as mean ± SD. TST, total sleep time; AHI, apnea-hypopnea index; CAI, central apnea index; ODI, 3% oxygen desaturation index; N1, N2, N3; R, sleep stages NREM 1, NREM 2, NREM 3, REM*.

CSR-CSA cycles were associated with 2,065 hyperpneic arousals. The percentage of CSR-CSA cycles without any hyperpneic arousal was 34 ± 16% (range: 8–56%). According to the classification scheme used in a previous study (4), in 25 ± 18% of the cycles (range: 5–70%) there was an “early-hyperpneic” arousal, i.e. arousal onset occurred very close to the beginning of hyperpnea (−0.4 ± 0.7 s). The duration of these arousals was 16.3 ± 7.6 s. Conversely, in 41 ± 15% of the cycles (range: 13–75%) there was a “late-hyperpneic” arousal, i.e. arousal onset occurred during the crescendo phase of hyperpnea (10.4 ± 2.1 s after the beginning of hyperpnea). Arousal duration was 9.8 ± 6.3 s. Both types of arousals were occasionally followed by 1 or 2 secondary arousals (4).

### Time Course of Arousal-Associated Changes in Ventilation

A total of 2,765 case-control breath pairs were identified and analyzed across patients. Valid AAC estimates were obtained in 8.2 ± 2.3 (range: 5–11) breaths of the hyperpnea. Breath duration was 3.2 ± 0.5 s and 3.4 ± 0.4 s in case and control breaths, respectively. The average difference in oxygen saturation between case and control breaths was −0.02 ± 0.21%. In most patients, the AAC could not be computed in the last part of the hyperpnea, owing to the fact that arousals only occasionally extended over or occurred during the decrescendo phase. Accordingly, results are presented up to 75% of hyperpnea length.

**Figure 5:**
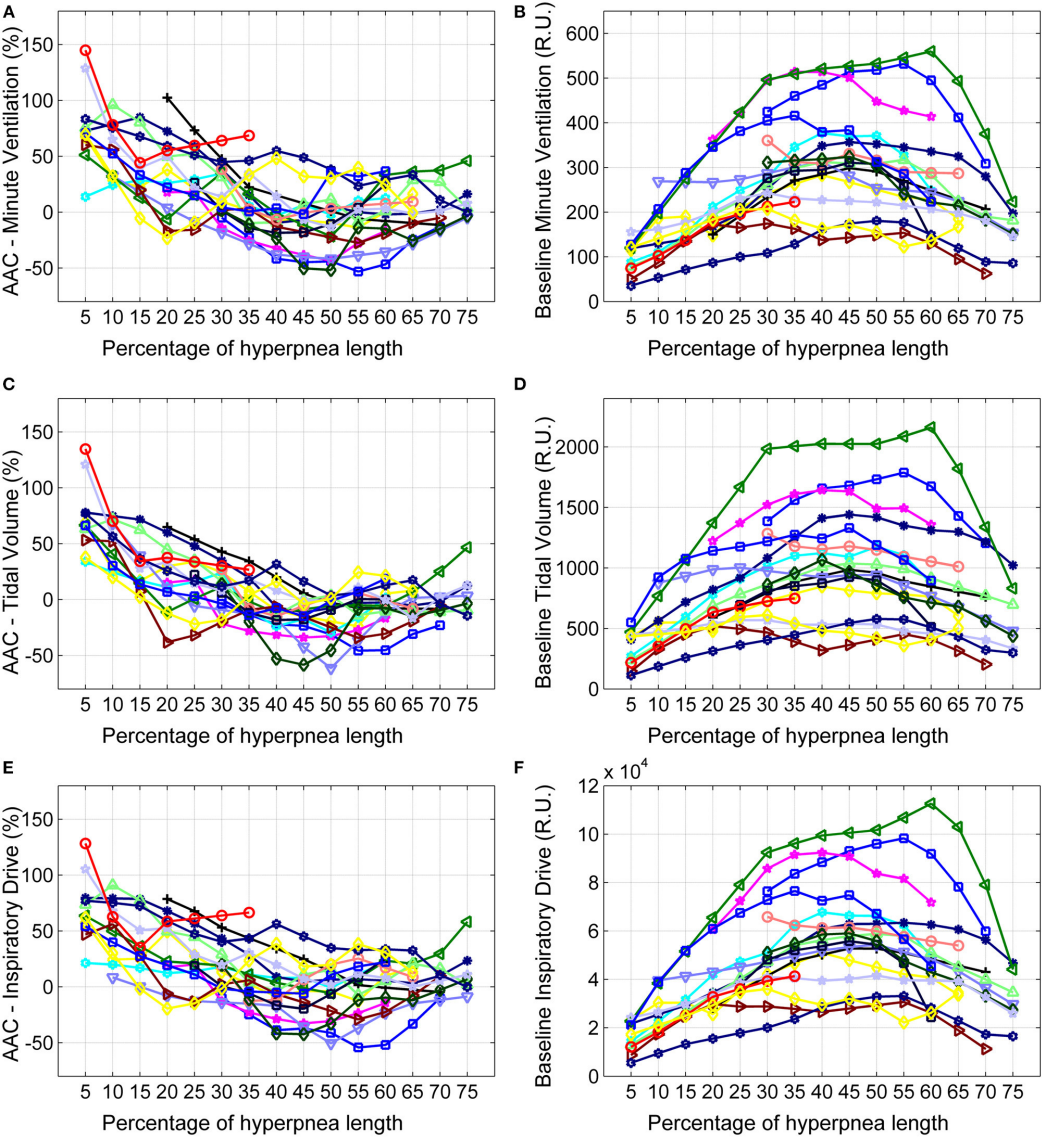
Left: individual time course of the arousal-associated change (AAC) in minute ventilation **(A)**, tidal volume **(C)** and inspiratory drive **(E)** as a function of the percentage of hyperpnea length. The AAC is expressed as a percentage of the average value of these parameters in the absence of arousals during hyperpneas (see equation 1). Right: individual time course of baseline values of minute ventilation **(B)**, tidal volume **(D)** and inspiratory drive **(F)** during hyperpneas. RU, relative units.

**Figure 6:**
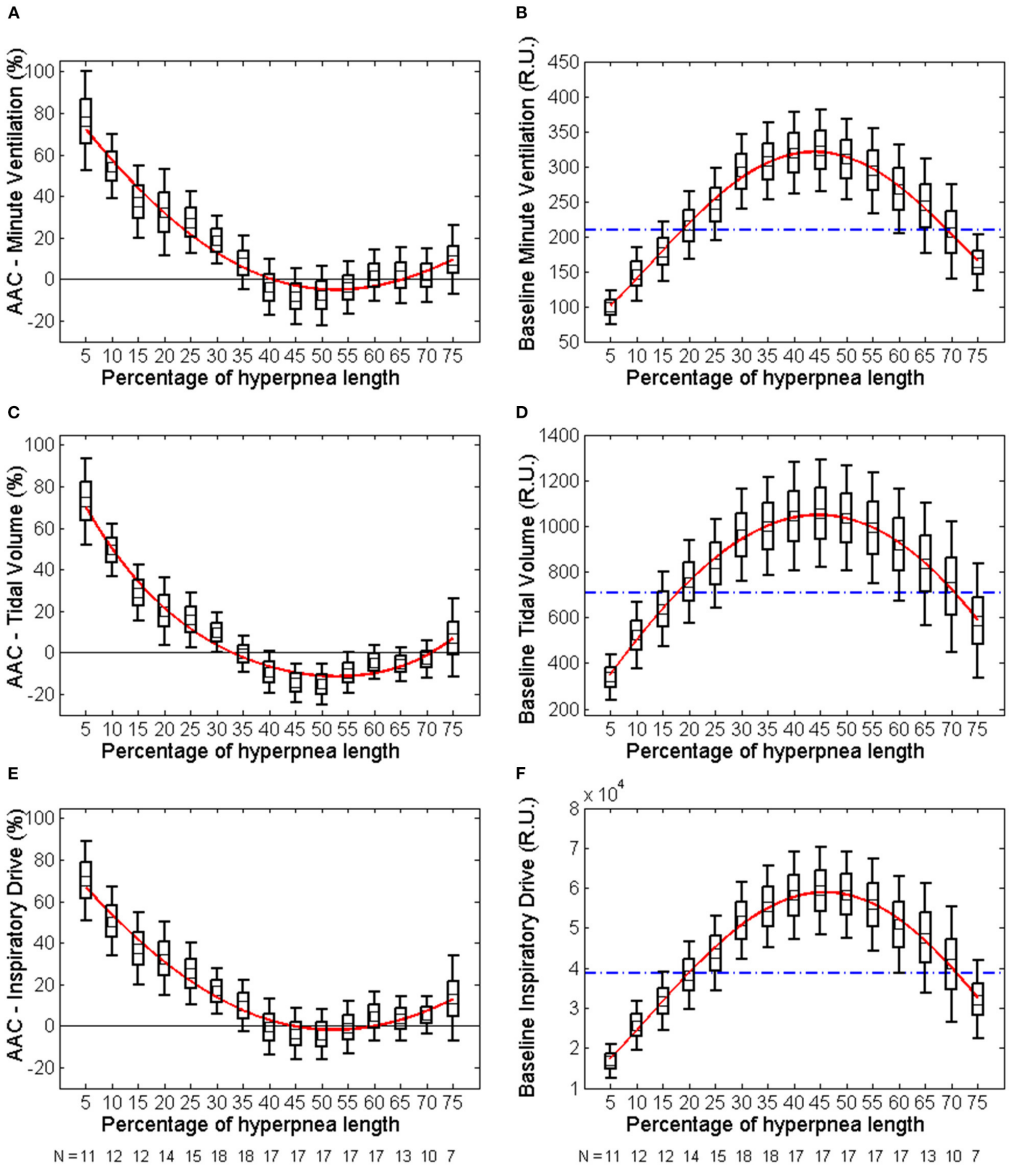
Left: descriptive statistics of the time course of the arousal-associated change (AAC) in minute ventilation **(A)**, tidal volume **(C)** and inspiratory drive **(E)** as a function of the percentage of hyperpnea length (middle point of each box: mean; box: mean ± SE; whiskers: mean ± 95% confidence interval). The AAC is expressed as a percentage of the average value of these parameters in the absence of arousals during hyperpneas (see equation 1). Whiskers not crossing the zero line indicate that the corresponding mean is significantly different from zero. Red solid lines are 4^th^-order polynomial fittings of mean values. Right: descriptive statistics of the time course of baseline values of minute ventilation **(B)**, tidal volume **(D)** and inspiratory drive **(F)** during hyperpneas. The blue dashed-dotted line is the mean normalization factor used for the estimation of the AAC in each patient (i.e., the denominator of equation 1). The numbers at the bottom of left and right panels indicate in how many patients the AAC could be estimated (at least 5 case-control pairs were required for each breath). RU, relative units.

The individual time course of the arousal-associated change (AAC) in ${\overset{∙}{V}}_{\text{I}}$, *V*_*T*_ and *ID* is plotted in Figures 5A,C,E. The individual time course of baseline values of the same ventilatory parameters is plotted in Figures 5B,D,F. Corresponding descriptive statistics are shown in Figure 6. The mean AAC in ${\overset{∙}{V}}_{\text{I}}$ (Figure 6A) was highest at the beginning of the hyperpnea (76.2 ± 35.3% of average ventilation in the absence of arousals, *p* < 0.0001 testing the null hypothesis AAC = 0), and then progressively shrank to reach a minimum (-8.4 ± 26.1%, *p* = 0.14) at 45% of hyperpnea length. Thereafter, there was a slight trend toward a positive change in ${\overset{∙}{V}}_{\text{I}}$ at 75% of hyperpnea length, which did not reach statistical significance (9.3 ± 17.7%, *p* = 0.21). Mean baseline ${\overset{∙}{V}}_{\text{I}}$ (Figure 6B) showed the typical crescendo-decrescendo pattern of Cheyne-Stokes respiration, with a peak at 45% of hyperpnea length. The correlation between the two time courses (AAC vs. baseline ${\overset{∙}{V}}_{\text{I}}$) was −0.82 (*p* = 0.0002).

Similar results were obtained for *V*_*T*_ and *ID* (Figures 5C–F, 6C–F). However, the three lowest AACs in *V*_*T*_ (45, 50, and 55% of hyperpnea length) were significantly lower than zero (*p* < 0.05 in all instances). The correlation between the time course of the AAC and that of baseline values was −0.88 (*p* < 0.0001) and−0.90 (*p* < 0.0001) for *V*_*T*_ and *ID*, respectively.

### Association Between Arousals and Post-hyperpnea Apnea Length

**Figure 7:**
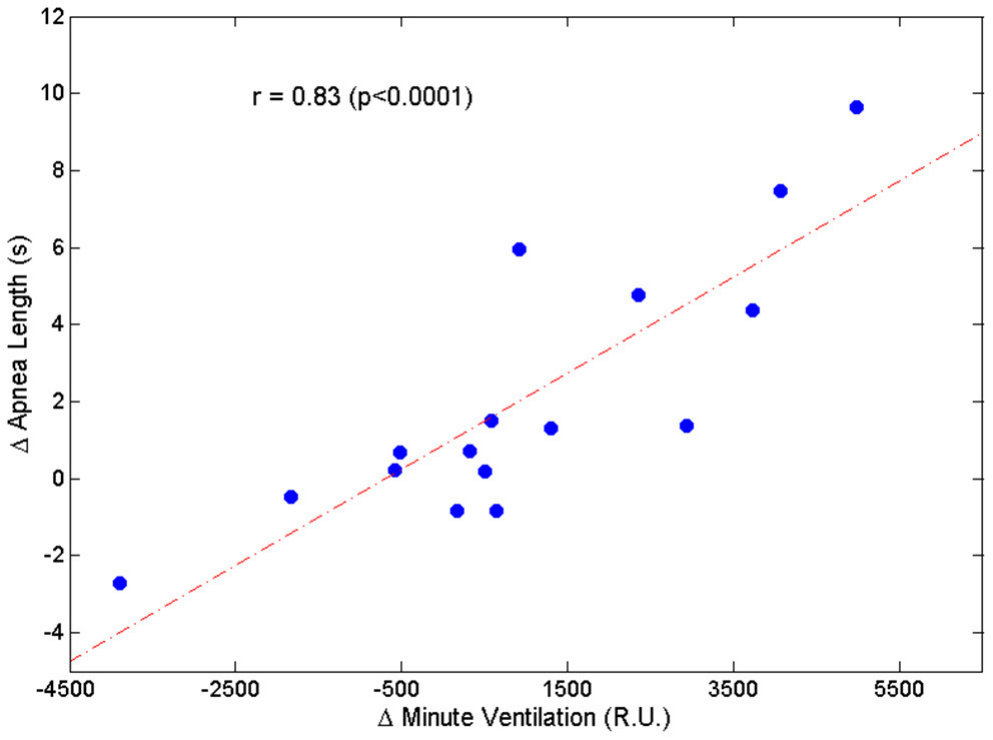
Relationship between the change in apnea length (Δ Apnea Length = average difference between the length of apneas preceded by a hyperpnea with arousal and the length of apneas preceded by a hyperpnea without arousal) and the corresponding change in mean ventilation (Δ Minute Ventilation = average difference between mean ventilation of hyperpneas with arousal and mean ventilation of hyperpneas without arousal).

Two patients were excluded from this analysis owing to insufficient number of case-control apnea pairs. Results are presented in Table 2. Apneas following a hyperpnea with arousal were 2.1 ± 3.4 s longer than those following a hyperpnea without arousal (*p* = 0.026), corresponding to a median percentage change of 4.6% (Q1, Q3: −0.7%, 20.5%; range: −8.5%, 36.2%) (*p* = 0.021). As shown in Figure 7, the change in apnea length was highly correlated with the corresponding change in mean ventilation of preceding hyperpneas (r = 0.83, *p* < 0.0001).

**Table 2:** Change in apnea length between apneas preceded by a hyperpnea with arousal (cases) and those preceded by a hyperpnea without arousal (controls).

	**Cases**	**Controls**	**Difference**	***p***
Apnea length (s)	25.7 ± 7.1	23.6 ± 6.2	2.1 ± 3.4	0.026
Hyperpnea length (s)	40.0 ± 9.2	41.8 ± 8.7	−1.8 ± 3.6	0.051
Cycle length (s)	65.7 ± 13.2	65.5 ± 13.1	0.2 ± 2.6	0.71
Mean SpO_2_ during the hyperpnea (%)	91.6 ± 2.6	91.6 ± 2.6	0.0 ± 0.1	0.25

*The corresponding change in hyperpnea and cycle length are also reported. Data are expressed as mean ± SD. RU, relative units*.

The change in apnea length was also correlated with the corresponding regional changes in mean ventilation, namely the region between 0 and 30% of hyperpnea length (r = 0.53, *p* = 0.036), and the region around peak hyperpnea (35–55% of hyperpnea length) (r = 0.67, *p* = 0.005). Moreover, it was also correlated with the incidence of early-hyperpneic arousals (r = 0.79, *p* = 0.0003). Using these three variables as explanatory variables, and the change in apnea length as the response variable, multiple linear regression analysis showed that mean ventilation in the region around peak hyperpnea and incidence of early-hyperpneic arousals were the only independent predictors of the change in apnea length (*p* = 0.004 and *p* = 0.015, respectively), with a coefficient of determination of 0.78.

## Discussion

The main findings of this study are: (1) arousals occurring during the hyperpneic phases of CSR-CSA are associated with a non-homogeneous change in ventilation, as a rapidly decreasing ventilatory overshoot occurs at the beginning of the hyperpnea, followed by a tendency toward a slight ventilatory undershoot around its peak; (2) the time course of this ventilatory change is highly correlated with that of baseline ventilation; (3) the presence of arousal during the hyperpnea is associated with a modest average increase in the length of the subsequent apnea, but there is a large inter-subject variability; (4) the incidence of early-hyperpneic arousals and the impact of arousals on ventilation in the region around peak hyperpnea are strong independent predictors of the change in post-hyperpnea apnea length.

### Interaction Between Arousals and Ventilation

We found that, at the resumption of breathing after an apnea, arousal from sleep was associated with a large ventilatory overshoot that rapidly declined in magnitude during the crescendo phase of hyperpnea. Around peak hyperpnea, approximately halfway into the hyperpnea, arousals tended to be associated with a slight ventilatory undershoot, and, thereafter, the trend reversed showing a slight, albeit not statistically significant, positive change in ventilation at 75% of hyperpnea length. These changes were closely inversely related to changes in baseline ventilation in the absence of arousals. These findings suggest that, on the one hand, the ventilatory overshoot associated with arousals involves only the initial portion of the hyperpnea, and, on the other hand, that the impact of arousals across the hyperpnea depends on the level of the underlying, chemoreflex-mediated ventilation.

Overall, there were only minor differences between the time course of changes in ${\overset{∙}{V}}_{\text{I}}$ and that of *V*_*T*_ and *ID*, except for a larger and statistically significant undershoot in *V*_*T*_ around peak hyperpnea.

### Possible Mechanisms Underlying the Time Course of Arousal-Associated Ventilatory Changes

Stimulation of chemoreceptors by combined hypoxia and hypercapnia promotes arousal directly, through neuronal connections from carotid body afferents and central respiratory chemoreceptors to the regions of the brain regulating arousal, and indirectly, through activation of the respiratory pattern generator in the brainstem (30). The latter activates wake-promoting neural pathways both directly and through peripheral reflex mechanisms secondary to the rise in ventilation (e.g., the lung inflation reflex) (30). Arousal, in turn, enhances the ventilatory response by recruiting descending pathways to the brainstem respiratory network (30). Many aspects of these long loop reflexes involving ascending and descending pathways have yet to be clarified, as well as the interaction between arousal, level of chemoreceptor stimulation and ventilation (14, 30), rendering interpretation of arousal-associated ventilatory changes during CSR-CSA difficult. This difficulty is further enhanced by the fact that, although high levels of chemoreceptor stimulation are typically required to produce arousal from sleep both in humans and in animal experiments (14), during CSR-CSA arousals may often occur at the resumption of breathing following apnea (4, 31), when the level of chemosensory stimuli is low, possibly due to a pathological hyperarousability induced by heart failure (32). Moreover, peripheral and central chemosensitivity is enhanced in patients with CSR-CSA (33), leading to a high respiratory drive during hyperpneas. We speculate that the large ventilatory overshoot observed in this study at the beginning of hyperpnea results predominantly from the excitatory effect of arousals on the respiratory pattern generator. As chemosensory stimuli increase during the crescendo phase, the observed progressive reduction of the extra-drive to breath provided by arousal (Figures 6A,C,E) suggests that the chemoreceptor-mediated excitatory drive progressively counteracts the excitatory drive from arousal networks, possibly through as-yet unclear interaction mechanisms and/or inhibitory effects mediated by lung stretch receptors. The latter hypothesis is supported by the high inverse correlation between the time course of the arousal-associated change in ventilation and that of baseline, chemoreceptor-driven ventilatory parameters. The fact that around peak hyperpnea there is a tendency toward a negative change in ventilation could be explained by the concurrent presence of a strong inhibitory feedback from lung receptors.

### Arousals and Change in Post-hyperpnea Apnea Length

The median increase in apnea length after hyperpneas with arousal was only about 5%, but there was a wide variability among patients with increases as large as about 36%. The change in apnea length was highly correlated with the concurrent change in mean ventilation across the hyperpnea, thus providing correlative evidence in support of the validity of the theoretical model predicting that arousals, by causing a transient ventilatory overshoot, set the stage for a longer apnea.

What could be the reason for the small median increase in apnea length? During CSR-CSA in the absence of arousals, PaCO_2_ increases and decreases cyclically (8, 22, 34). During its decrescendo phase, the apnea threshold is crossed and central apnea ensues (23). The greater the magnitude of the hyperpnea, the lower the minimum value reached by PaCO_2_ and the longer the length of the apnea. Thus, although any increase in ventilation during the crescendo phase of hyperpnea would contribute to lower PaCO_2_, the magnitude of peak hyperpnea would be the critical factor determining the minimum value of PaCO_2_. Therefore, one reason for the modest average impact of arousals on apnea length may be the fact that ventilation tends to decrease around peak hyperpnea (Figure 6A). Another reason may be that some patients have a low incidence of early hyperpneic arousals and, therefore, the likelihood of developing a ventilatory overshoot is low. A third reason may be the fact that in some patients the magnitude of the arousal-associated change in ventilation in the first breaths of the hyperpnea is low (Figure 5A). Using information about these factors in a multivariate linear regression model, we found that the incidence of early-hyperpneic arousals and the arousal-associated change in ventilation in the region around peak hyperpnea were the only independent predictors of the change in apnea length. Therefore, depending on the level of these 2 factors in each patient, quite different changes in apnea length might occur, thus explaining the large variability in observed results.

### Implications

Although, given its observational nature, the present study cannot provide evidence of a causal relationship between arousals, ventilatory overshoot and increase in post-hyperpnea apnea length, the soundness of the methodology used in the analysis and the consistency and strength of results strongly support this possibility. Accordingly, our results suggest that pharmacological manipulation of the arousal threshold to prevent the occurrence of arousals may represent a therapeutic option to reduce/suppress breathing instability in selected patients with heart failure and CSR-CSA. Since prospective trials are needed to test this therapeutic strategy, the study findings provide valuable information for the identification of patients suitable for inclusion in these studies.

### Limitations of the Study

Because it is not possible to establish a clear causal link between arousal, ventilatory overshoot and change in post-hyperpnea apnea length, we have assessed the validity of this conceptual model using observational data (i.e., polysomnographic recordings during CSR-CSA) and a case-control approach, as often performed in clinical research (35). Therefore, the study findings provide only indirect evidence supporting causality.

## Conclusions

This study provides correlative evidence that arousals produce a large ventilatory overshoot during the rising phase of the hyperpnea and tend to cause a slight ventilatory undershoot around its peak. On average, the impact of arousals on post-hyperpnea apnea length is modest but varies widely among patients. The incidence of early-hyperpneic arousals and the arousal-associated change in ventilation around peak hyperpnea are strong and independent determinants of the change in apnea length. These findings suggest that preventing arousals might represent a therapeutic option for reducing/suppressing CSR-CSA in appropriately selected patients.

## Data Availability Statement

The raw data supporting the conclusions of this article will be made available by the authors, without undue reservation.

## Ethics Statement

Ethical review and approval was not required for the study on human participants in accordance with the local legislation and institutional requirements. The patients/participants provided their written informed consent to participate in this study.

## Author Contributions

GP conceived the study, conducted data analysis, and wrote the manuscript. ER conducted experiments, carried out the analysis of sleep studies, and contributed to data interpretation. CB and ML reviewed the manuscript and provided critical feedback. RM contributed to the design of the study, developed the software for signal analysis, and contributed to writing the manuscript. All authors contributed to the article and approved the submitted version.

## Conflict of Interest

The authors declare that the research was conducted in the absence of any commercial or financial relationships that could be construed as a potential conflict of interest.

## Publisher's Note

All claims expressed in this article are solely those of the authors and do not necessarily represent those of their affiliated organizations, or those of the publisher, the editors and the reviewers. Any product that may be evaluated in this article, or claim that may be made by its manufacturer, is not guaranteed or endorsed by the publisher.
